# Supplementary material for: Combined models of violent conflict and natural hazards improve predictions of household mobility in Bangladesh
Source: Commun Earth Environ. 2025 Dec 19;7(1):67. doi: 10.1038/s43247-025-03086-3 (PMC12823410; doi:10.1038/s43247-025-03086-3)
Supplement: Supplementary file 2 — Supplementary information to: Combined models of violent conflict and natural hazards improve predictions of household mobility in Bangladesh [file 43247_2025_3086_MOESM2_ESM.pdf]

Supplementary information to: Combined models of violent conflict and natural hazards improve predictions of household mobility in Bangladesh

Contents

1 Supplementary Methods **2**

1.1 Data Description 2

1.2 Descriptive statistics: Dependent Variable 5

1.3 Coding procedure: Dependent Variable 8

1.4 Model specification 9

1.4.1 Features Baseline model 9

1.4.2 Features Violence model 11

1.4.3 Features Disasters/Hazards model 13

1.5 Estimation and evaluation strategy 14

2 Supplementary Analysis **16**

2.1 Predictive Performance and Maps 16

2.2 SHAP Analysis 19

2.3 Regression results 23

1 Supplementary Methods

1.1 Data Description

Table S1: List of survey questions used to construct the dependent variable measuring household migration.

Bangladesh Integrated Household Survey questions				
Target variable	File name in survey wave 1	File name in survey wave 2	File name in survey wave 3	Variable label in the survey waves
Mobility	h041-mod-v1-male	h053-r2-mod-v1-male	h072-bihs-r3-male-mod-v1	v1-01: Has anyone, who was a members of your household, migrated in the past five years;
Mobility	h041-mod-v1-male	h053-r2-mod-v1-male	h072-bihs-r3-male-mod-v1	v1-03: When did they migrate? (year);
Mobility	h041-mod-v1-male	h053-r2-mod-v1-male	h072-bihs-r3-male-mod-v1	v1-04: When did they migrate? (month);
Mobility	h041-mod-v1-male	h053-r2-mod-v1-male	h072-bihs-r3-male-mod-v1	v1-10: If in-country, write Zila code;
Mobility	h041-mod-v1-male	h053-r2-mod-v1-male	h072-bihs-r3-male-mod-v1	v1-11: If abroad, write country code;

Table S2: List of survey questions used to construct the household level features

Bangladesh Integrated Household Survey questions				
Features in the Baseline model	File name in survey wave 1	File name in survey wave 2	File name in survey wave 2	Variable label in survey waves
Religion	h001-mod-a-male	h001-r2-mod-a-male	h001-r3-mod-a-male	a13: Household Head's religion: 1 (Muslim), 2 (Hindu) and 3 (Christian);
Language	h001-mod-a-male	h001-r2-mod-a-male	h001-r3-mod-a-male	a14: Primary language spoken;
Ethnicity	h001-mod-a-male	h001-r2-mod-a-male	001-r3-mod-a-male	a15: Household's Ethnic group: 1 (Bangali), 2 (Bihari) and 3 (Tribal);
Sex	003-mod-b1-male	h003-r2-mod-b1-male	h010-bihs-r3-male-mod-b1	b1-01: Household's head sex: 1 (Male) and 2 (Female);
Literacy	003-mod-b1-male	h003-r2-mod-b1-male	h010-bihs-r3-male-mod-b1	b1-07: Literacy of the member;
Education	003-mod-b1-male	h003-r2-mod-b1-male	h010-bihs-r3-male-mod-b1	t1-02(14): Education (highest class passed) of the member;
Occupation	003-mod-b1-male	h003-r2-mod-b1-male	h010-bihs-r3-male-mod-b1	b1-10: Current main occupation (as binary for agricultural or non/agricultural occupations);
Mobile asset value	006-mod-d1-male	h010-r2-mod-d1-male	h015-bihs-r3-male-mod-d1	d1-10: current value/ if asset sold today how much will you receive?;
Mobile asset proportion of ownership	006-mod-d1-male	h010-r2-mod-d1-male	h015-bihs-r3-male-mod-d1	d1-05: what portion of asset owned by household?;
Savings	008-mod-e-male	h012-r2-mod-e-male	h017-bihs-r3-male-mod-e	e06: Total amount currently saved in this place;
Access to loans	009-mod-f-male	h013-r2-mod-f-male	h018-bihs-r3-male-mod-f	f02: Does any adult in the household currently have a loan with any individual or institution?;
Land size	010-mod-g-male	h014-r2-mod-g-male	h020-bihs-r3-male-mod-g	g01: plot type (cultivable/arable land; pasture; cultivable pond=1; otherwise=0);
Monthly expenditure (value)	hhexpenditures-r123	hhexpenditures-r123	hhexpenditures-r123	pc-expm: Per capita hh expenditure/month (including house rent and use of value of durables);
Agricultural subsidies	022-mod-j2-male	h033-r2-mod-j2-male	h040-bihs-r3-male-mod-j2a	j2-01: Do you have an agriculture input subsidy card?;
Livestock (value)	h023-mod-k1-male		h043-bihs-r3-male-mod-k1	k1-02b: What was livestock's value?
Income (value)	h044-mod-v4-male	h056-bihs-r2-male-mod-v4	h076-bihs-r3-male-mod-v4	v4-01-v4-13: overall income received
Incoming remittances (value)	h042-mod-v2-male	h054-r2-mod-v2-male	h073-bihs-r3-male-mod-v2	v2-06: How much money in total did your household receive in last 12 months?
Benefited from safety net program	h040-mod-u-male	h054-r2-mod-v2-male	h073-bihs-r3-male-mod-v2	v2-06: Have you gotten any assistance from a safety net program?
Active member	h073-mod-weai-we-male	h095-r2-weai-ind-mod-we4-male	h084-bihs-r3-male-weai-ind-mod-we4	e07a - e07k : Are you an active member of various groups?
Active leader	h073-mod-weai-we-male	h095-r2-weai-ind-mod-we4-male	h084-bihs-r3-male-weai-ind-mod-we4	e08a - e08k: do you have a leadership position in various groups?
Satisfied with relationships	h077-mod-weai-wf02-wf11-male	h105-r2-weai-ind-mod-we6b-male	h089-bihs-r3-male-weai-ind-mod-we6b	wf04c: How satisfied you are (within 10 scales) your contacts with friends or relative?
Satisfied with alternative place	h077-mod-weai-wf02-wf11-male	h105-r2-weai-ind-mod-we6b-male	h089-bihs-r3-male-weai-ind-mod-we6b	wf04d: how satisfied you are (within 10 scales) your possibilities of going to other places?
Satisfied with life	h077-mod-weai-wf02-wf11-male	h105-r2-weai-ind-mod-we6b-male	h089-bihs-r3-male-weai-ind-mod-we6b	wf04f: how satisfied you are (within 10 scales) your satisfaction with your life overall?
Own mobile	$006_{m}od_{d}1_{m}ale$	$h010_{r}2_{m}od_{d}1_{m}ale$	$h015_{b}ihs_{r}3_{m}ale_{m}od_{d}1$	d1-03: Does your household own the item?
Own radio	$006_{m}od_{d}1_{m}ale$	$h010_{r}2_{m}od_{d}1_{m}ale$	$h015_{b}ihs_{r}3_{m}ale_{m}od_{d}1$	d1-03: Does your household own the item?
Own tv	$006_{m}od_{d}1_{m}ale$	$h010_{r}2_{m}od_{d}1_{m}ale$	$h015_{b}ihs_{r}3_{m}ale_{m}od_{d}1$	d1-03: Does your household own the item?

Table S3: Results of two-sided t-tests comparing households that dropped out of the sample with those that remained. The table reports p-values, showing statistically significant differences for most variables between the two groups.

Sub-samples			p-Value
	Remained in sample	Dropped records	
Religion			
Muslim	0.891	0.840	< 0.01
Christian	0.002	0.001	< 0.01
Hindu	0.105	0.159	< 0.01
Ethnicity			
Bangali	0.996	1.00	< 0.01
Other ethnicity	0.003	0.000	< 0.01
Literacy	0.464	0.503	< 0.01
Education			
Primary	0.246	0.281	< 0.01
Secondary	0.242	0.266	< 0.01
Tertiary	0.015	0.024	0.05
No educ	0.496	0.429	< 0.01
Age of hh head			
Between 18 and 24	0.038	0.030	< 0.01
Between 25 and 34	0.234	0.168	< 0.01
Between 35 and 48	0.376	0.419	< 0.01
Between 49 and 70	0.321	0.344	< 0.01
Over 71	0.028	0.039	0.01
Gender hh head			
Female	0.185	0.134	0.01
Male	0.814	0.866	0.01
Other			
Migration history	0.046	0.023	< 0.01
Received remittances	0.002	0.001	< 0.01
Mobile assets	38235	34732	< 0.01
Machinery assets	2816	3253	< 0.01
Savings	14868	12227	< 0.01
Loans	33699	21978	< 0.01
Type of land	0.440	0.505	< 0.01
Size of land	89	103	< 0.01
Agricultural subsidies	0.077	0.135	< 0.01
Livestock value	16072	19471	< 0.01
Benefited from social policies	0.447	0.543	< 0.01
Income level	3969	1750	< 0.01

1.2 Descriptive statistics: Dependent Variable

Table S4: Number and percentage of households reporting migration events by year. The table presents the share of households that reported at least one migration event in each survey year. Across the full sample, only about 4.5% of households report a migration event in a given year, showing the rarity of the outcome and the challenge of predicting household-level migration.

Year	HH w. international mig.	HH w. internal mig.	HH w. any mig.	Share HH w. any mig. (%)
2011	72	203	275	5.00
2012	37	7	44	0.80
2013	50	120	170	3.09
2014	38	135	173	3.14
2015	34	95	129	2.34
2016	82	386	468	8.50
2017	76	310	386	7.01
2018	86	353	439	7.98

Table S5: Number and percentage of surveyed households by district. The table summarizes the distribution of surveyed households across districts in Bangladesh, showing both absolute counts and relative percentages for each district included in the analysis.

District Name	N. Households	Share (%)
Comilla	117	5.61
Chandpur	102	4.89
Chittagong	94	4.51
Sunamganj	93	4.46
Mymensingh	71	3.41
Tangail	71	3.41
Sylhet	65	3.12
Habiganj	65	3.12
Noakhali	61	2.93
Lakshmipur	54	2.59
Feni	53	2.54
Netrakona	52	2.50
Kishoreganj	49	2.35
Bhola	48	2.30
Barisal	47	2.26

Continued on next page

Table S5: Number and percentage of surveyed households by district. The table summarizes the distribution of surveyed households across districts in Bangladesh, showing both absolute counts and relative percentages for each district included in the analysis.

District Name	N. Households	Share (%)
Sherpur	45	2.16
Naogaon	44	2.11
Faridpur	44	2.11
Bogra	40	1.92
Kurigram	38	1.82
Narsingdi	37	1.78
Maulvibazar	36	1.73
Gaibandha	32	1.54
Narayanganj	31	1.49
Cox'S Bazar	31	1.49
Pirojpur	30	1.44
Gopalganj	30	1.44
Bagerhat	29	1.39
Brahamanbaria	28	1.34
Patuakhali	27	1.30
Natore	27	1.30
Manikganj	26	1.25
Satkhira	25	1.20
Munshiganj	24	1.15
Khulna	24	1.15
Jhalokati	23	1.10
Jamalpur	23	1.10
Madaripur	22	1.06
Dinajpur	21	1.01
Pabna	20	0.96

Continued on next page

Table S5: Number and percentage of surveyed households by district. The table summarizes the distribution of surveyed households across districts in Bangladesh, showing both absolute counts and relative percentages for each district included in the analysis.

District Name	N. Households	Share (%)
Nawabganj	18	0.86
Lalmonirhat	17	0.82
Rangpur	16	0.77
Shariatpur	16	0.77
Sirajganj	16	0.77
Jessore	15	0.72
Dhaka	15	0.72
Nilphamari	15	0.72
Narail	14	0.67
Joypurhat	14	0.67
Kushtia	13	0.62
Barguna	13	0.62
Bandarban	13	0.62
Gazipur	13	0.62
Jhenaidah	12	0.58
Chuadanga	10	0.48
Rajbari	10	0.48
Thakurgaon	9	0.43
Rajshahi	9	0.43
Rangamati	9	0.43
Khagrachhari	6	0.29
Meherpur	4	0.19
Magura	4	0.19
Panchagarh	4	0.19

1.3 Coding procedure: Dependent Variable

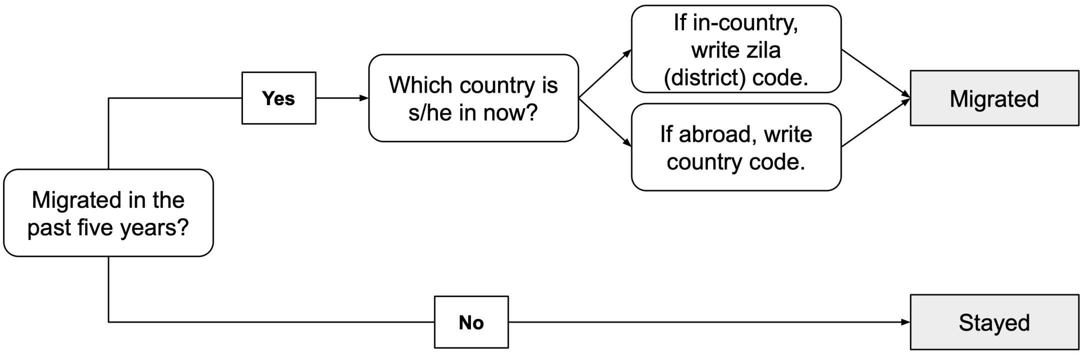

Figure S1: Coding of the dependent variable based on survey questions in the Bangladesh Integrated Household Survey (BIHS). The figure illustrates the steps used to construct the household-level migration variable from the BIHS questionnaire items.

1.4 Model specification

1.4.1 Features Baseline model

Table S6: Summary statistics of features included in the Baseline model before imputation. Non-logged values are included as reference. The table reports descriptive statistics for all variables derived from the Bangladesh Integrated Household Survey (BIHS), Nighttime Light (NTL) data, and the Cross-National Data of Sub-National Violence (xSub) dataset.

feature	intuitive name	source	count	mean	std	min	max
nlight_mean	Nightlight	NTL	44024	6.032	4.266	0	44.750
pg_gcp_ppp_li	Gross cell product and purchasing power parity	xSub	44024	3.990	2.054	0.35	10.004
head_age	Head age of hh	BIHS	44024	47.399	14.215	17	102.000
head_sex	Head sex of hh	BIHS	44024	1.185	0.389	1	2.000
prim_educ	Primary education	BIHS	42967	0.259	0.438	0	1.000
sec_educ	Secondary education	BIHS	42967	0.262	0.440	0	1.000
ter_educ	Tertiary education	BIHS	42967	0.019	0.138	0	1.000
no_educ	No education	BIHS	42967	0.459	0.498	0	1.000
religion	Religion	BIHS	44024	1.111	0.322	1	3.000
ethnicity	Ethnicity	BIHS	44024	1.006	0.110	1	3.000
head_ocup_agg	Head occupation of hh	BIHS	44024	0.389	0.488	0	1.000
mig_history	Migration history	BIHS	44024	0.062	0.241	0	1.000
ln_mob_asset_value_own	Ln (mobile asset value)	BIHS	16509	9.924	2.407	0	15.281
mob_asset_value_own	mobile asset value	BIHS	16509	65907	117852	0	4328400
ln_savings	Ln (savings)	BIHS	16509	5.300	4.643	0	15.607
savings	savings	BIHS	16509	23545	102246	0	6000000
ln_loans	Ln (loans)	BIHS	16509	6.900	5.046	0	16.002
loans	loans	BIHS	16509	53033	154292	0.00	8905000
ln_land_size	Ln (land size)	BIHS	16509	3.459	1.649	0	8.037
land_size	land size	BIHS	16509	89	144	0	3092
ln_pc_expm	Ln (monthly expenditure value)	BIHS	26643	7.998	0.526	6.304	10.394
pc_expm	monthly expenditure value	BIHS	26643	3449	2186	545	32663
ag_subsidy	Ln (agricultural subsidy)	BIHS	16509	0.104	0.305	0	1.000
ln_livestock_value	Ln (livestock value)	BIHS	33018	6.531	4.246	0	13.885
livestock_value	livestock value	BIHS	33018	19370	38546	0	1072000
sl_benefited	Safety net program	BIHS	16509	0.44	0.448	0	1.000
ln_income	Ln (income)	BIHS	16509	1.059	2.940	0	15.520
income	income	BIHS	16509	3295	48244	0	5500000
ln_remittances_in_value	Ln (received remittances)	BIHS	26644	3.199	4.884	0	15.202
remittances_in_value	received remittances	BIHS	26644	24436	83789	0	4000000
active_member	Active member	BIHS	36576	0.147	0.354	0	1.000
active_leader	Active leader	BIHS	36576	0.034	0.182	0	1.000
satisfied_leave	Satisfied with alt places	BIHS	22120	5.833	2.277	1	10.000
satisfied_life	Satisfied with life	BIHS	22120	6.904	2.278	1	10.000
mobile	Mobile	BIHS	41176	0.842	0.365	0	1.000
radio	Radio	BIHS	41176	0.026	0.158	0	1.000
tv	TV	BIHS	41176	0.305	0.452	0	1.000

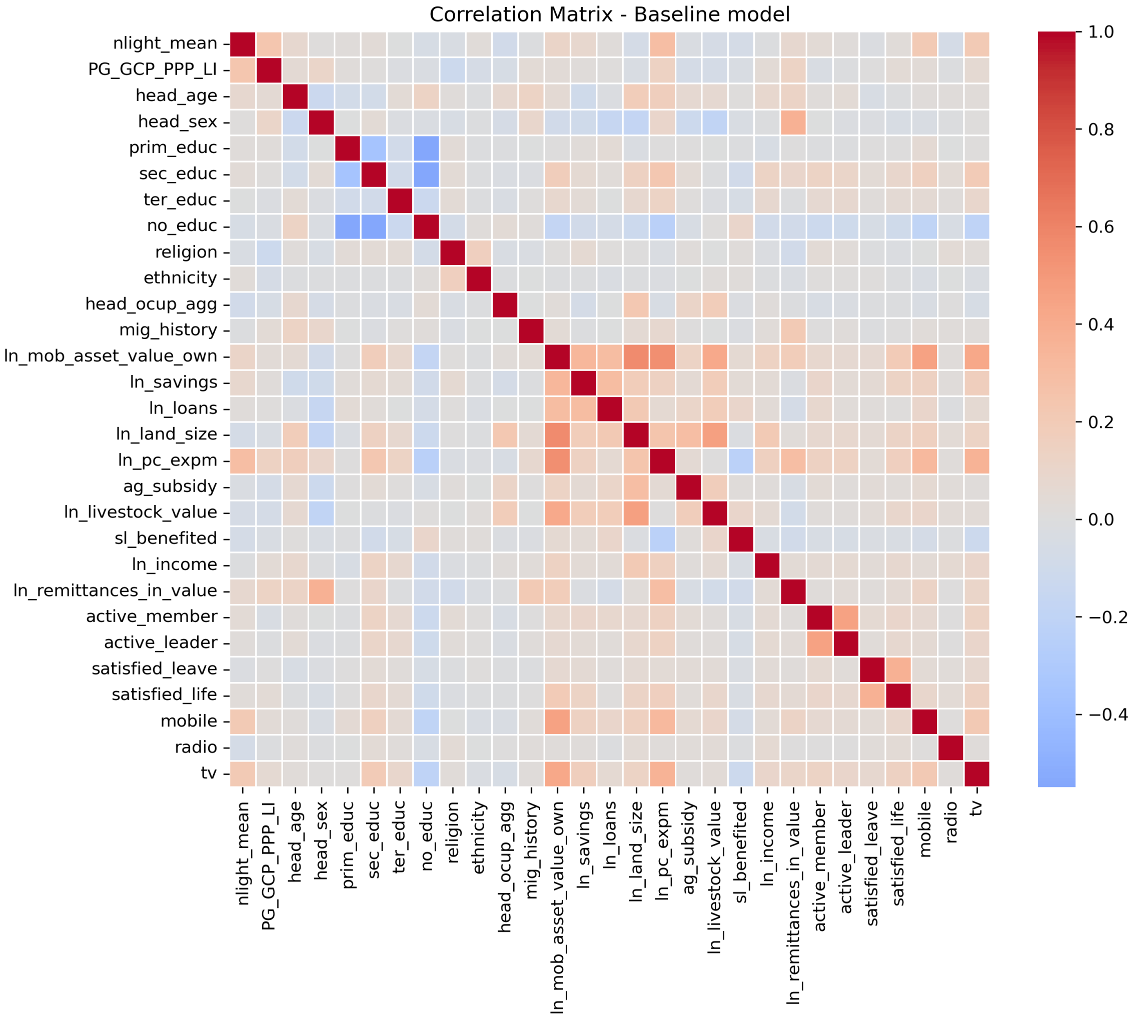

Figure S2: Correlation matrix of variables in the Baseline model. The figure shows pairwise correlation coefficients among the model variables. Darker colors represent stronger correlations, with red indicating positive and blue indicating negative relationships.

1.4.2 Features Violence model

Table S7: Summary statistics of features included in the Violence model, in addition to the Baseline features. The table reports descriptive statistics for all variables derived from the Uppsala Conflict Data Program (UCDP) Georeferenced Events Dataset, the Armed Conflict Location and Event Data Project (ACLED), and the Deadly Electoral Conflict Dataset (DECO)

feature	intuitive name	source	count	mean	std	min	max
ged_best_ns_tlag12	Sum NS violence (t-12)	UCDP	44024	0.121	0.652	0.000	8
ged_best_sb_tlag12	Sum SB violence (t-12)	UCDP	44024	0.203	1.416	0.000	21
ged_best_osv_tlag12	Sum OSV violence (t-12)	UCDP	44024	0.089	0.846	0.000	20
ged_best_ns_splag1_tlag12	Sum NS violence spatial lag(t-12)	UCDP	44024	0.514	1.500	0.000	10
ged_best_sb_splag1_tlag12	Sum SB violence spatial lag (t-12)	UCDP	44024	1.064	3.975	0.000	30
ged_best_osv_splag1_tlag12	Sum OSV violence spatial lag (t-12)	UCDP	44024	0.546	2.448	0.000	21
acled_prrio_count_tlag12	Count protests with riots (t-12)	ACLED	44024	13.291	22.775	0.000	312
acled_prrio_count_splag1_tlag12	Count protests with riots spatial lag (t-12)	ACLED	44024	67.897	73.972	0.000	452
acled_prex_count_tlag12	Count protests with excessive violence (t-12)	ACLED	44024	0.801	2.262	0.000	37
acled_prex_count_splag1_tlag12	Count protests with excessive violence spatial lag (t-12)	ACLED	44024	4.464	6.763	0.000	46
ged_best_ns_ts_decay_6_tlag12	NS violence decay (t-12)	UCDP	44024	0.103	0.264	0.000	1
ged_best_osv_ts_decay_6_tlag12	OSV violence decay (t-12)	UCDP	44024	0.059	0.212	0.000	1
ged_best_sb_ts_decay_6_tlag12	SB violence decay (t-12)	UCDP	44024	0.045	0.196	0.000	1
acled_prrio_count_ts_decay_6_tlag12	Protests with riots decay (t-12)	ACLED	44024	0.980	0.107	0.000	1
acled_prrio_count<br>_splag1_ts_decay_6_tlag12	Protests with riots decay spatial lag (t-12)	ACLED	44024	1.000	0.003	0.891	1
acled_prex_count_ts_decay_6_tlag12	Protests with excessive violence decay (t-12)	ACLED	44024	0.407	0.445	0.000	1
acled_prex_count<br>_splag1_ts_decay_6_tlag12	Protests with excessive violence decay spatial lag (t-12)	ACLED	44024	0.847	0.312	0.000	1
deco_best_ns_tlag12	Sum NS electoral violence (t-12)	DECO	44024	0.343	5.857	0.000	137
deco_best_sb_tlag12	Sum SB electoral violence (t-12)	DECO	44024	0.145	0.979	0.000	16
deco_best_osv_tlag12	Sum OSV electoral violence (t-12)	DECO	44024	0.135	1.206	0.000	20
deco_best_ns_splag1_tlag12	Sum NS electoral violence spatial lag (t-12)	DECO	44024	2.228	15.262	0.000	140
deco_best_sb_splag1_tlag12	Sum SB electoral violence spatial lag (t-12)	DECO	44024	0.775	2.548	0.000	16
deco_best_osv_splag1_tlag12	Sum OSV electoral violence spatial lag (t-12)	DECO	44024	0.770	3.151	0.000	23
deco_best_ns_ts_decay_6_tlag12	NS electoral violence decay (t-12)	DECO	44024	0.085	0.247	0.000	1
deco_best_osv_ts_decay_6_tlag12	OSV electoral violence decay (t-12)	DECO	44024	0.047	0.193	0.000	1
deco_best_sb_ts_decay_6_tlag12	SB electoral violence decay (t-12)	DECO	44024	0.072	0.237	0.000	1

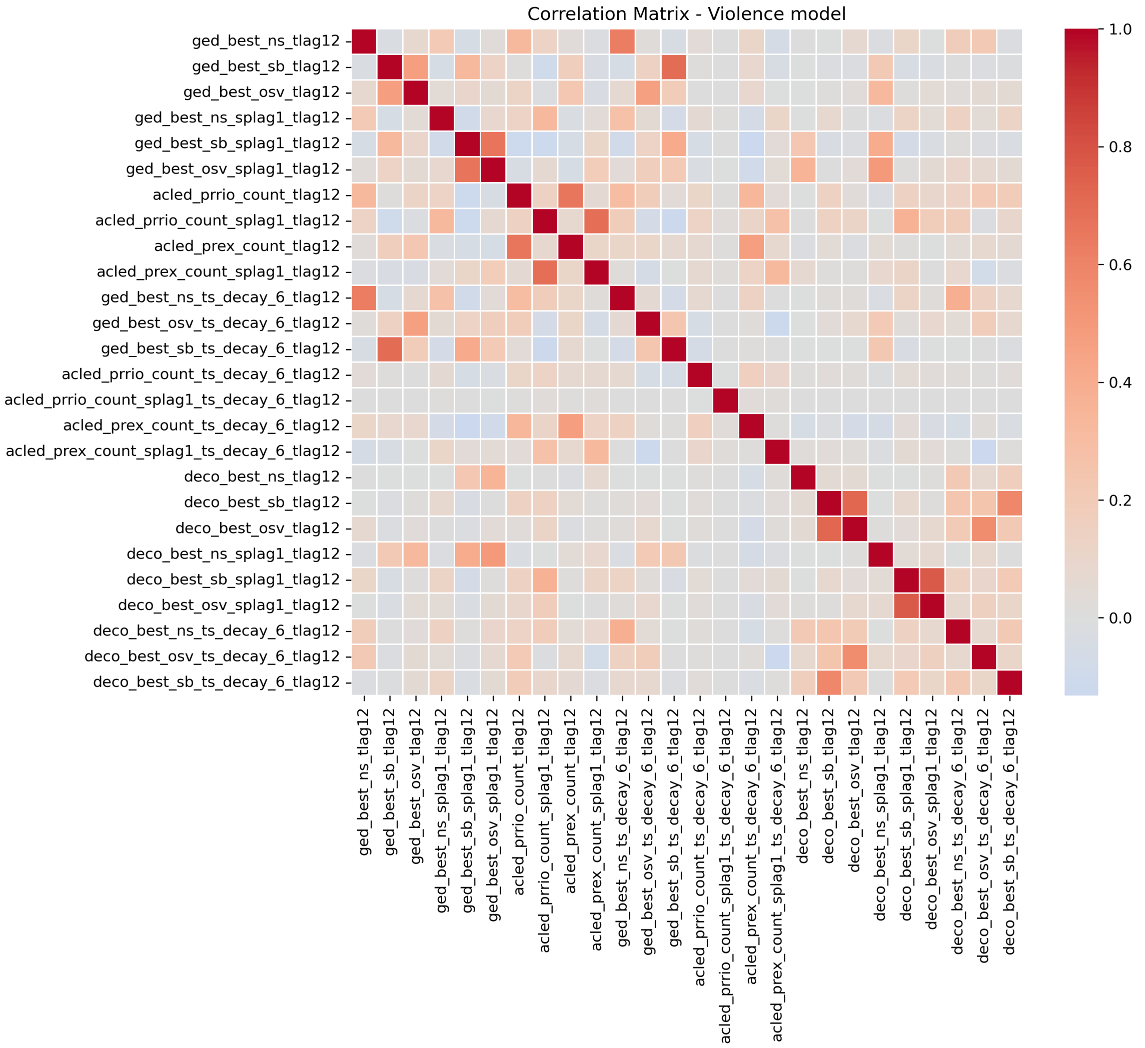

Figure S3: Correlation matrix of variables in the Violence model. The heatmap shows pairwise correlation coefficients among all variables included in the model. Darker colors represent stronger correlations, with red indicating positive and blue indicating negative relationships

1.4.3 Features Disasters/Hazards model

Table S8: Summary statistics of features included in the Disasters/Hazards model, in addition to the Baseline features. The table reports descriptive statistics for all variables derived from the Geocoded Disasters (GDIS) Dataset, the Dartmouth Flood Observatory (DFO), and the Standardized Precipitation–Evapotranspiration Index (SPEI) database.

feature	intuitive name	source	count	mean	std	min	max
gdis_n_disasters_tlag12	Count of disaster events (t-12)	GDIS	44024	1.020	1.247	0.000	7.000
flood_dummy_ts_decay_6_tlag12	Flood decay (t-12)	DFO	44024	0.617	0.403	0.000	1.000
gdis_disastertype_flood_ts_decay_6_tlag12	Flood decay (t-12)	GDIS	44024	0.382	0.406	0.000	1.000
gdis_disastertype_landslide_ts_decay_6_tlag12	Landslide decay (t-12)	GDIS	44024	0.083	0.240	0.000	1.000
gdis_disastertype_storm_ts_decay_6_tlag12	Storm decay (t-12)	GDIS	44024	0.296	0.391	0.000	1.000
spei_3_severe_ts_decay_6_tlag12	Drought over 3 months decay	SPEI	44024	0.474	0.422	0	1

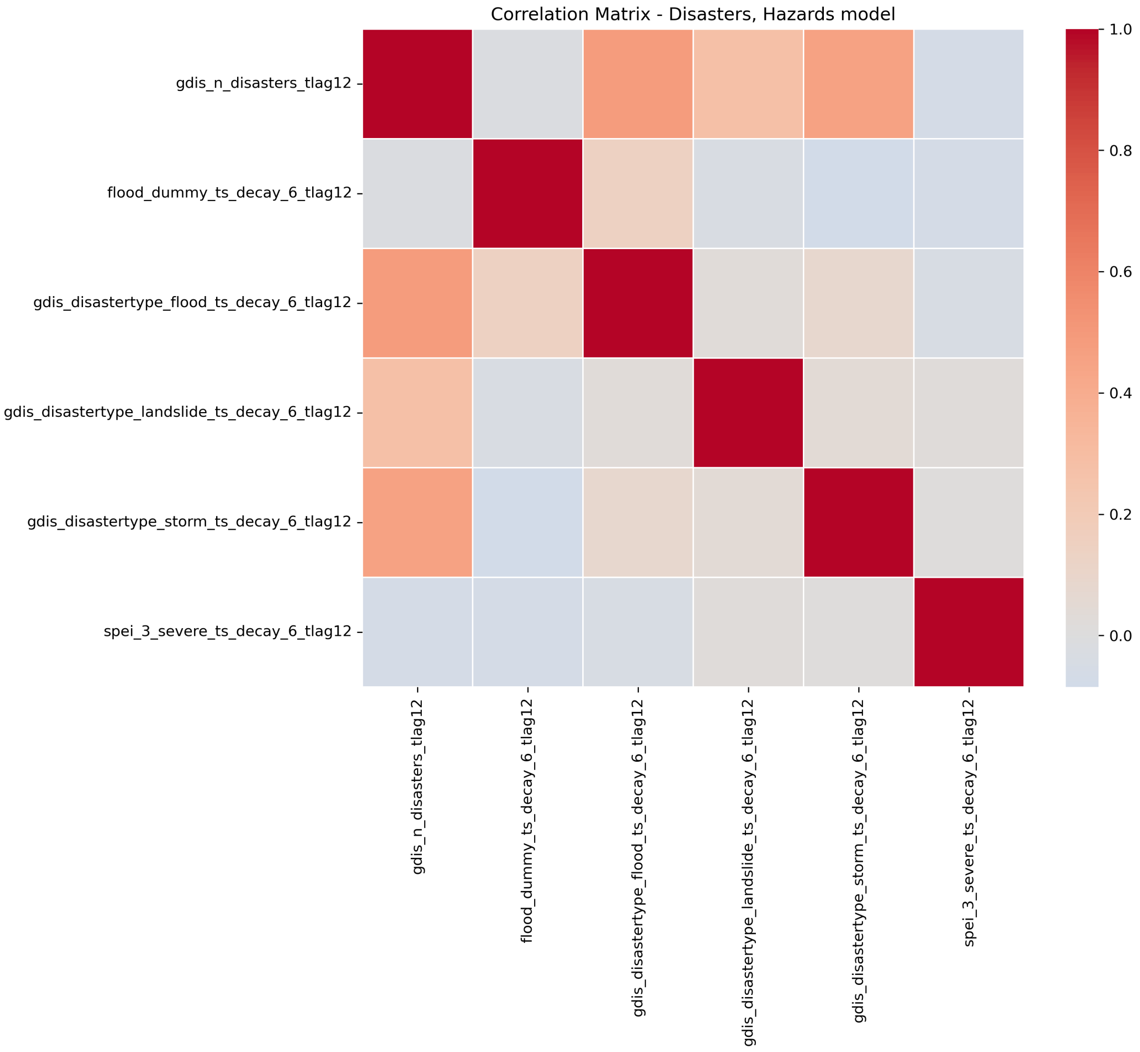

Figure S4: Correlation matrix of Disasters/Hazards variables. The figure visualizes the pairwise correlation coefficients among key variables in the model. Darker shades indicate stronger correlations, with red representing positive correlations and blue indicating negative correlations.

Table S9: Number of households affected by violence, disaggregated by violence type and year. The table includes total violence exposure (all types combined) and individual counts for each type of violence, including state-based, non-state, and one-sided violence.

Year	any violence	ns (GED)	sb (GED)	osv (GED)	ns (DECO)	sb (DECO)	osv (DECO)
2011	400	280	60	60	0	0	0
2012	0	0	0	0	0	0	0
2013	320	140	0	0	240	0	0
2014	2903	1903	0	360	780	1060	380
2015	443	40	0	0	223	403	80
2016	1143	60	60	443	200	400	603
2017	1980	0	520	640	720	140	320
2018	1020	0	920	100	0	0	0

Table S10: Number of households affected by disasters, disaggregated by violence type and year. The table includes total disaster exposure (all types combined) and individual counts for each type of violence, including landslides, storms, and floods.

Year	any disaster	landslides	storms	floods
2011	1580	100	840	860
2012	2700	960	840	1460
2013	2680	0	2580	820
2014	460	0	460	0
2015	1600	0	700	1500
2016	3623	960	1520	1920
2017	2940	0	1780	1360
2018	3620	300	560	3100

1.5 Estimation and evaluation strategy

The models reported in the main article are estimated using extreme gradient boosting. The *learning rate* and *maximum depth* for each model specification are tuned and specified with help of an 'early stopping routine'. Hyper-parameter tuning in combination with early stopping aims at reducing over-fitting and enhancing predictive accuracy. The implemented routine for each model includes the following steps:

1. Specify a range of possible values for the parameters of interest, i.e. a maximum depth ranging between 2 and 6 and a learning rate of 0.001, 0.003, 0.01, 0.03 and 0.1.

2. Set the number of trees to n = 250 and the early stopping rounds to n = 25.

3. Split the data into two folds, including a training (80%) and test (20%) set.

4. Run the model specifications for each possible parameter specification and save information

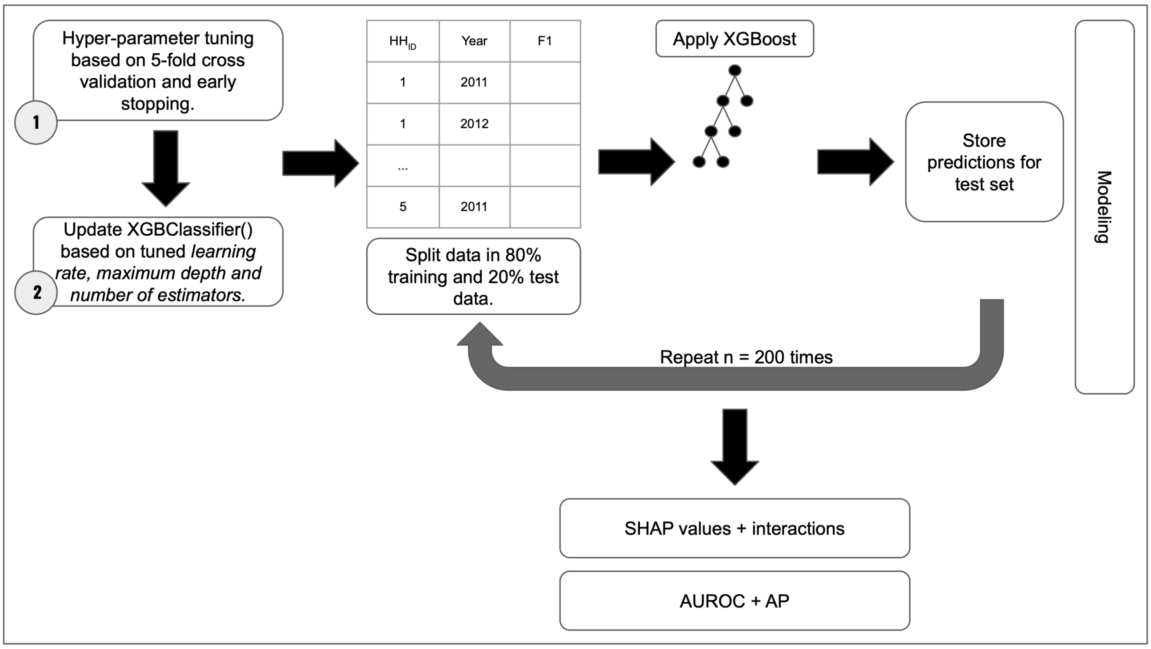

Figure S5: Graphical representation of the estimation and evaluation framework. The figure illustrates the workflow used to train, test, and validate model performance across 250 repetitions, outlining the main analytical steps and data flow.

about 1) the difference between the average precision of the training and test set, 2) the average precision for the test set, and 3) the optimal number of trees based on early stopping.

5. Repeat steps number 3. and 4. five times and store the mean, maximum and minimum across all repetitions.

6. Specify the parameter values of the final model based on the combination of values that minimises the trade-off between over-fitting and predictive accuracy.

Table S11 shows the tuned parameter specification for each model specification included in the analysis.

Table S11: (Tuned) hyper-parameter specifications.

	n trees	maximum depth	learning rate	n features
Baseline	250	2	0.03	29
Violence	250	2	0.03	55
Disasters/Hazards	250	2	0.03	36
Violence & Disasters/Hazards	250	3	0.03	62

For the computation of the SHAP values, we rely on the tree-based implementation (TreeSHAP) using the *shap* library. This method is computationally superior to other approximation algorithms and is supported by "theoretical guarantees of local accuracy and consistency" [1]. A distinct advantage of TreeSHAP is its ability to decompose the impact of a feature into two components: the primary SHAP value, which quantifies the individual contribution of a feature, and the SHAP interaction value, which assesses the joint contribution of feature pairs to the prediction. This decomposition allows for a more nuanced understanding of feature interactions within the model [1].

2 Supplementary Analysis

2.1 Predictive Performance and Maps

Table S12 reports F1-scores for each model across 250 repetitions. For comparison, we also report the in-sample performance of the logistic regression models shown in Figures S14(d), S15(b) and S15(c). These values reflect in-sample fit, since the regression models are primarily included to assess whether coefficient directions align with the SHAP analyses as a robustness check. As expected, the XGBoost models outperform the logistic regressions, as shown in Table S.13, and we note that out-of-sample performance of the regressions would likely be worse. Low F1-scores at the 0.5 threshold are expected in this rare-event setting, but at the 0.1 threshold the Full Distress model achieves clear improvements over the Baseline as well as over the logistic models.

Table S12: Average F1-scores across 250 repetitions. The table reports macro F1-scores at probability thresholds of 0.1 and 0.5. Macro F1 assigns equal weight to each class, providing a balanced measure of model performance.

Model	Macro F1 (0.1)	Macro F1 (0.5)
Baseline	0.559	0.488
Violence	0.577	0.488
Disasters/Hazards	0.576	0.488
Violence & Disasters/Hazards	0.579	0.488

Political insecurity and climate-related hazards and disasters occur locally and vary across regions in Bangladesh. In Figure S6, we present AP scores aggregated to the first-order division level for the Baseline model (a), as well as the differences in AP scores between the Baseline

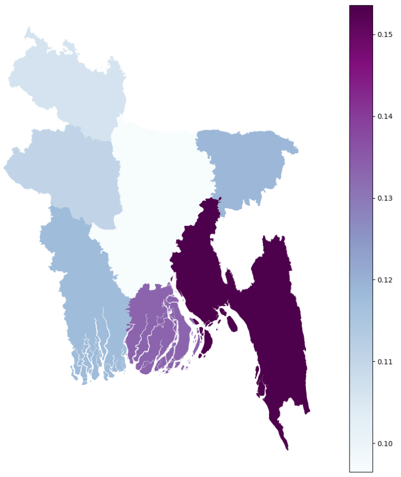

(a)

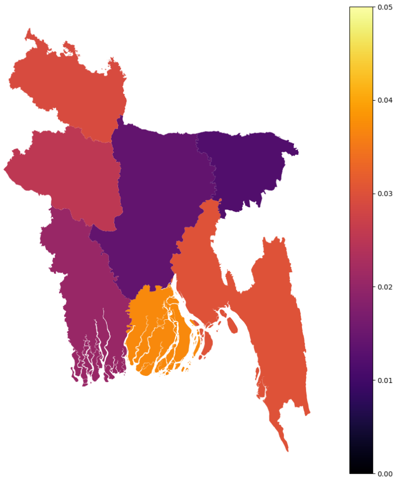

(b)

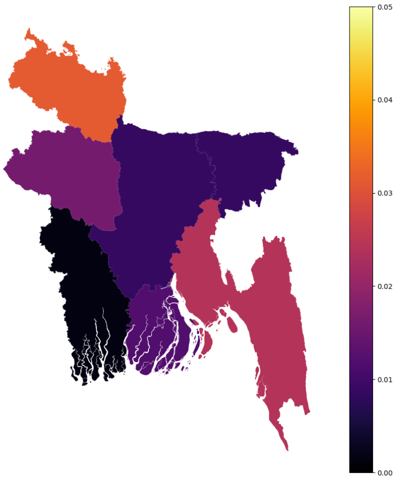

(c)

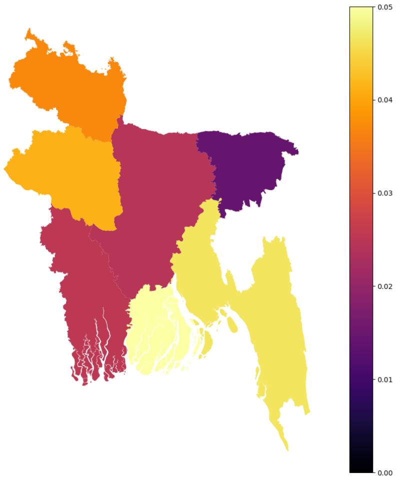

(d)

Figure S6: Mapping of aggregated average AP scores to the first-order division level for (a) Baseline model and the differences in AP scores between Baseline and (b) Violence, (c) Disasters/Hazards, and (d) Full Distress models across divisions in Bangladesh. The maps use pre-2015 administrative boundaries and therefore do not show Mymensingh as a separate division.

Table S13: In-sample performance metrics for logistic regression models (see Supplementary Figures S14(d), S15(b) and S15(c).

Model	Macro F1 (0.5)	Macro F1 (0.1)	AUROC	Average Precision (AP)
Electoral Violence (decay)	0.488	0.543	0.678	0.087
Disasters (decay)	0.488	0.548	0.683	0.091
Electoral violence & Disasters (decay)	0.488	0.550	0.698	0.096

and each thematic model (b-d). The Baseline model performs best in the southeastern regions (Chittagong and Barisal) while displaying the lowest performance for Dhaka. The performance of the Violence model increases across all regions with the highest improvements in Barisal in the South and Rangpur in the North (indicated by orange colors). The smallest improvements are observed for the Sylhet and Dhaka divisions (purple colors). Similar patterns are seen for the Hazards/Disasters model, however, with overall smaller increases in predictive performance. Notable improvements are observed for Chittagong (in the South), alongside Barisal and Rangpur. Finally, in line with the performance evaluated in the main text, the Full Distress exhibits the most significant positive changes in AP scores across regions highlighting the importance of accounting for interactions between features of the Violence and the Disasters/Hazards models. This is further supported by the case of Rajshahi, where the combined model demonstrates a considerable improvement, indicative of households facing multiple hazards simultaneously.

2.2 SHAP Analysis

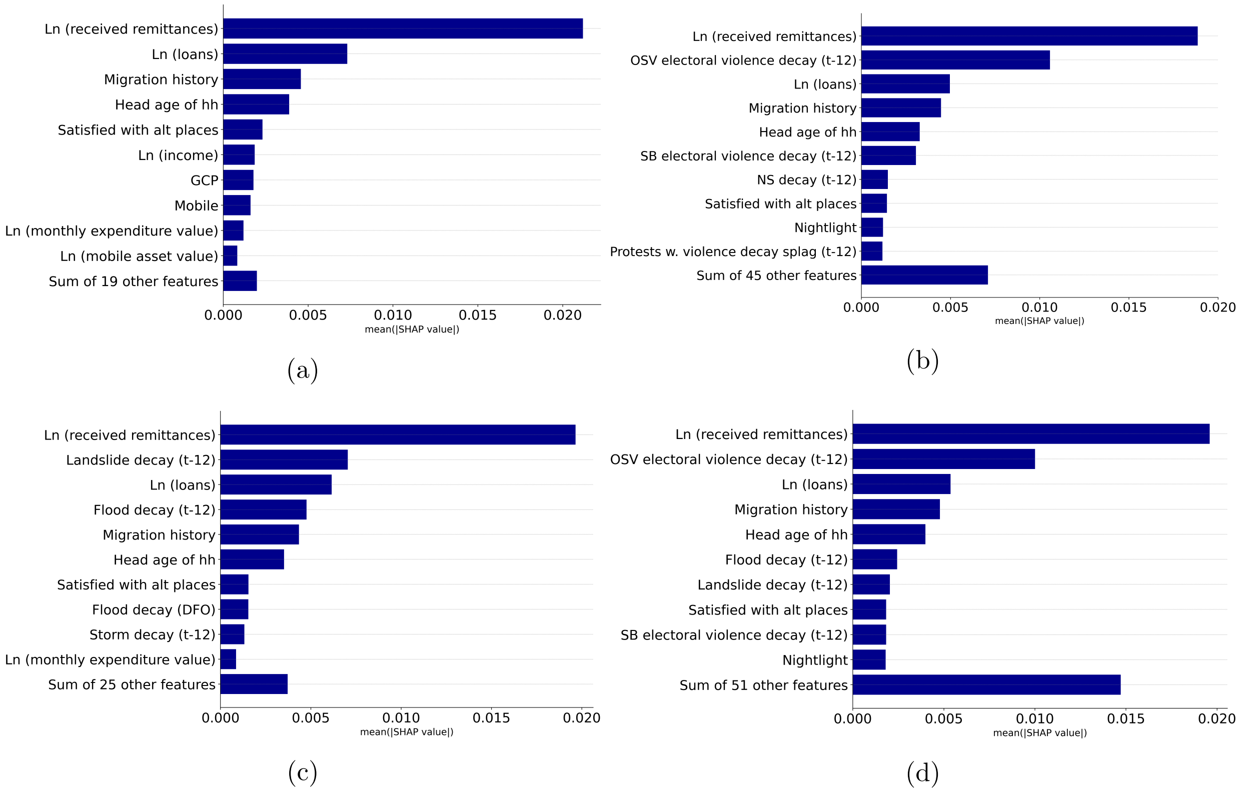

Figure S7: Mean absolute SHAP values, expressed as probabilities, for all models: (a) Baseline, (b) Violence, (c) Disasters/Hazards, and (d) Full Distress. The figure shows the individual feature rankings based on averaged mean absolute SHAP values for each model. The x-axis represents the average impact of each feature on the model output, calculated across all dataset observations and averaged over 100 repetitions. The y-axis lists features in order of decreasing importance. "Sum of features" aggregates the SHAP values of the remaining features in each model. Features related to violence and natural hazards are lagged by 12 months (t–12).

Figure S9 shows the Shapley values obtained by pooling and sampling across repetitions with above-median out-of-sample accuracy (AP score) only, while Figure S10 presents the corresponding cohort analysis of feature effects under the same restriction. In both cases, the resulting patterns are very similar to those in Figure 4 and Figure 5 in the main text, demonstrating that the observed effects are robust to excluding poorly performing folds.

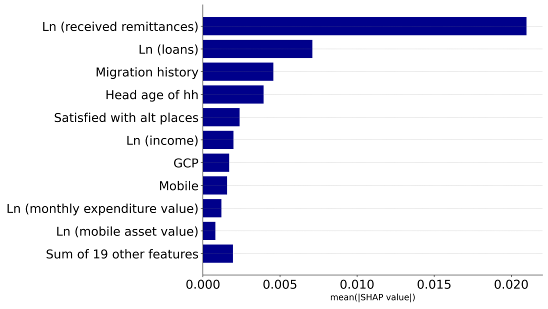

(a)

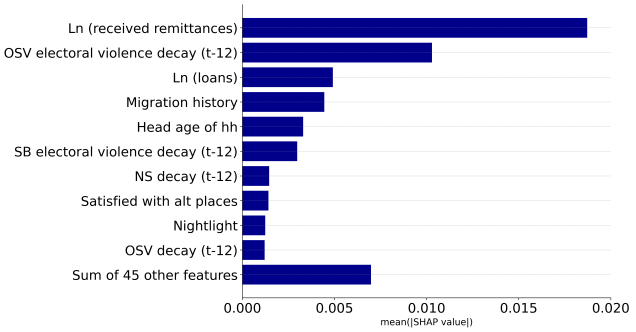

(b)

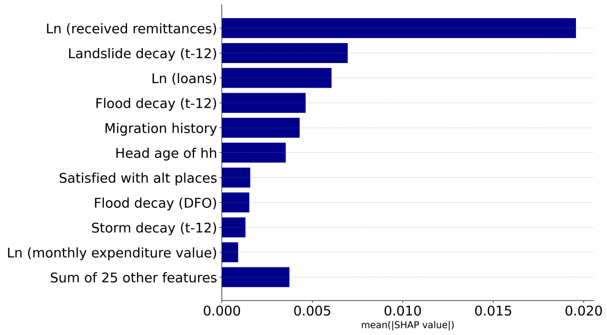

(c)

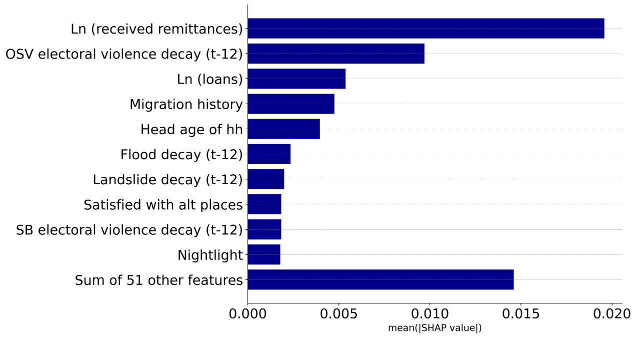

(d)

Figure S8: Mean absolute SHAP values, expressed as probabilities, for all models: (a) Baseline, (b) Violence, (c) Disasters/Hazards, and (d) Full Distress. The figure shows feature rankings based on mean absolute SHAP values across repetitions with above-median out-of-sample accuracy. The x-axis represents the average impact of each feature on the model output, calculated over all dataset observations across these high-performing repetitions. The y-axis lists features in order of decreasing importance. "Sum of features" aggregates the SHAP values of the remaining features in each model.

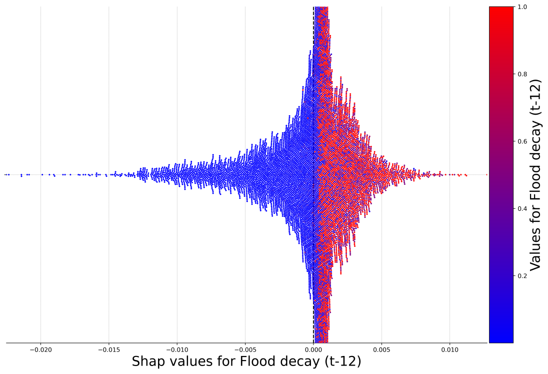

(a)

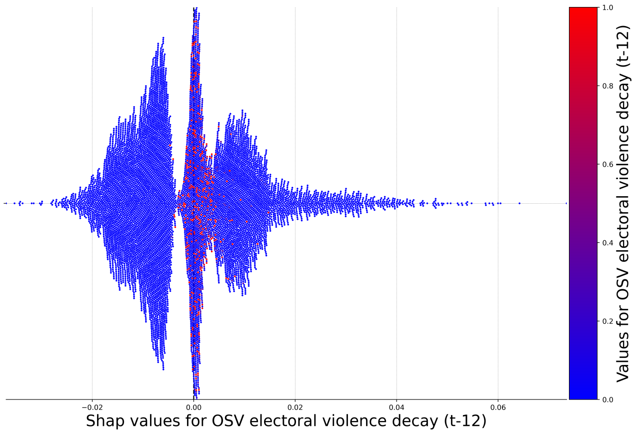

(b)

Figure S9: The x-axis shows individual Shapely values (probabilities) from the *Full Distress* model for individual observations of a) time since flooding decay and b) time since one-sided electoral violence decay, all lagged by 12 months (t-12). Each dot is an observation. The y-axis shows data point distribution, presenting the spread of data points at various Shapley values. The dot colors indicate actual variable values, red for high and blue for low values (see color-bar).

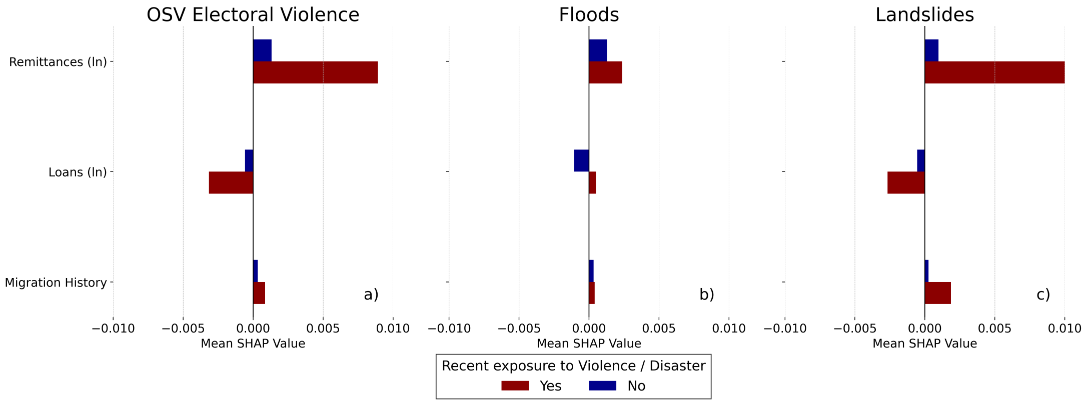

Figure S10: Mean SHAP values, expressed as probabilities, for the cohort analysis of recent exposure to one-sided electoral violence, floods and landslides in the *Full Distress* model. The x-axis represents the mean net SHAP value for each feature including remittances, loans and migration history of the households with (red color) and without (blue color) recent exposure, averaged across repetitions with above-median out-of-sample accuracy.

Figure S11 offers initial insights into the potential presence of interactions, with darker colors indicating a stronger interaction effect between the two features. The most pronounced interaction is observed between one-sided electoral violence and landslides on one hand, and one-sided and state-based electoral violence on the other. Additionally, we see interactions, yet to a smaller degree, between one-sided violence and other types of natural-hazard-related disasters including droughts. For state-based and non-state electoral violence, the most notable effects are seen for floods. It is also noteworthy that some interaction is evident between different types of natural-hazard-related disasters, especially when looking at floods. Among this category, the most substantial interaction occurs between floods and landslides, highlighting their inherent connection and mutually reinforcing effects.

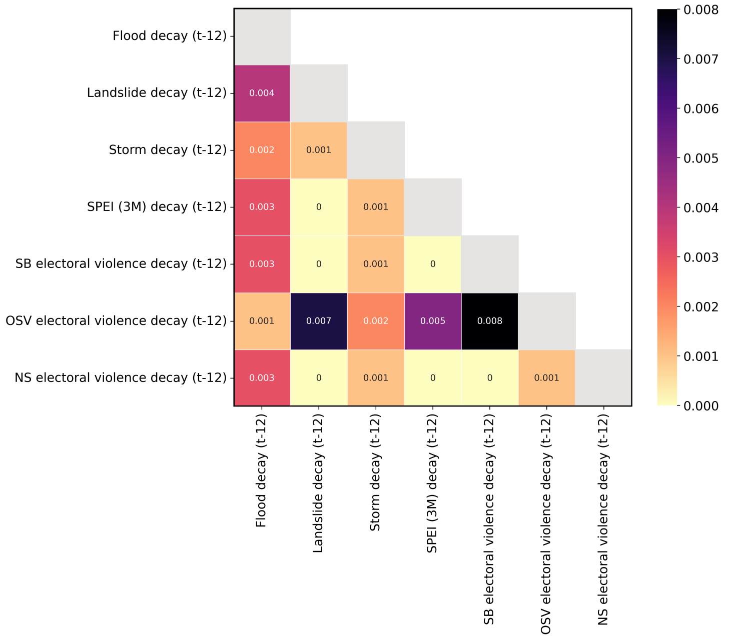

Figure S11: Interactions measured as absolute SHAP interaction values (in log-odds) between different forms of violent outcomes and climate-related disasters based on the *Full Distress* model, averaged across all repetitions. The darker the color, the higher the interaction.

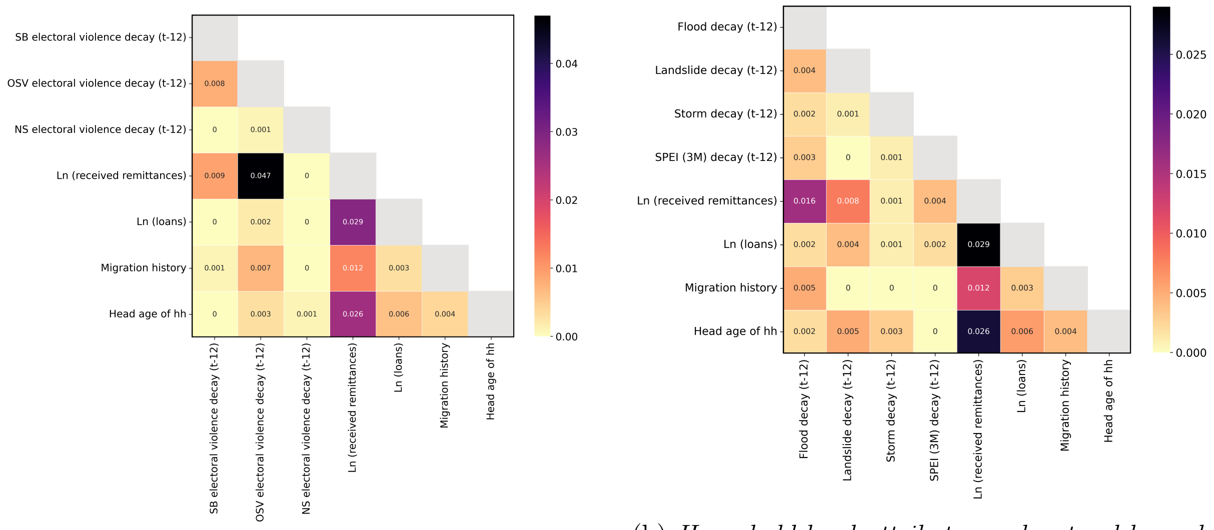

(a) *Household-level attributes and violence.*

(b) *Household-level attributes and natural-hazard-related disasters.*

Figure S12: Interactions measured as absolute SHAP interaction values (in log-odds) between household-level attributes and different forms of violent outcomes as well as climate-related disasters based on the *Full Distress* model, averaged across repetitions with above-median out-of-sample accuracy. The darker the color, the higher the interaction.

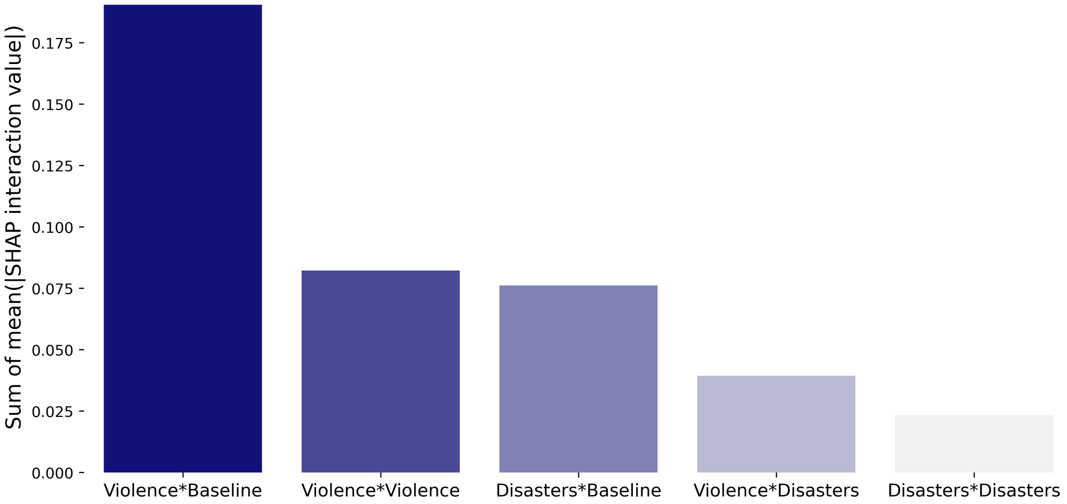

Figure S13: Sum of mean absolute SHAP interaction values (in log-odds) between feature groups of violence, hazards/disasters and household characteristics, analyzed through the *Full Distress* model. The y-axis indicates the average impact across the dataset, averaged across repetitions with above-median out-of-sample accuracy.

2.3 Regression results

To further validate the results of the main analysis and underscore the advantages of employing a machine learning approach, Figures S14 and S15 display the outcomes from a mixed-effect logistic regression with random intercepts at the district level. Due to multicollinearity, the models presented here rely solely on a subset of features included in the XGBoost models. We use the following equation for the full model, shown in Figure 15 c):

$$logit(\Pr(y_{i,t}=1)) = \alpha + u_i + \beta_1 Violence_{i,t-12} + \beta_2 Hazard_{i,t-12} + \gamma^\top \mathbf{H}_{i,t} + \delta^\top \mathbf{D}_{i,t}, \quad (1)$$

where i indexes districts (Admin 2) and t indexes years. The term $u_i \sim \mathcal{N}(0, \sigma_u^2)$ is a district-year random intercept capturing unobserved time-invariant heterogeneity. $Violence_{i,t-12}$ and $Hazard_{i,t-12}$ denote the 12-month lagged (t–12) counts and time-decayed measures of conflict events and natural hazards, respectively. $X_{i,t}$ is a vector of time-varying controls that includes both household- and district-level covariates. We estimate several specifications that differ only in the operationalization of $Violence_{i,t-12}$ and $Hazard_{i,t-12}$, corresponding to Figure S.14–S.15.

Upon comparing the estimates of the household-level features from the regression models with the SHAP summary plots in Figure S16, notable similarities emerge in terms of the importance

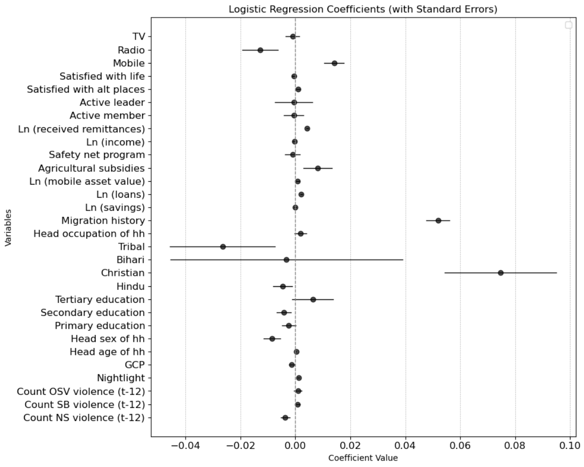

(a)

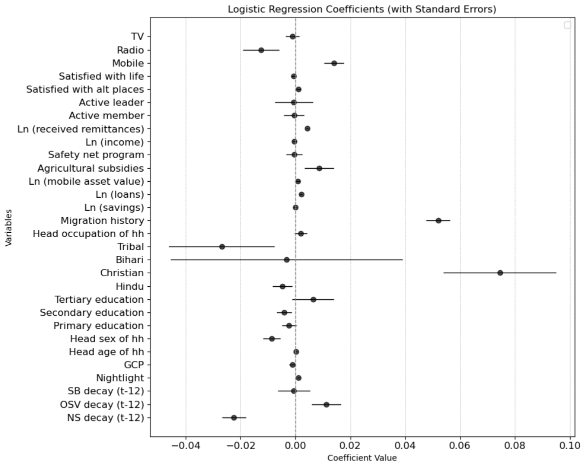

(b)

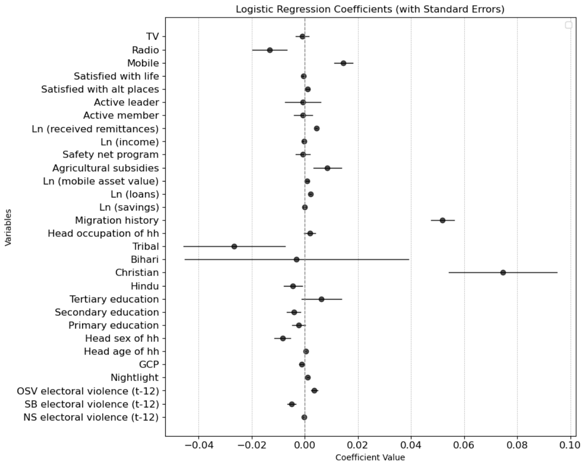

(c)

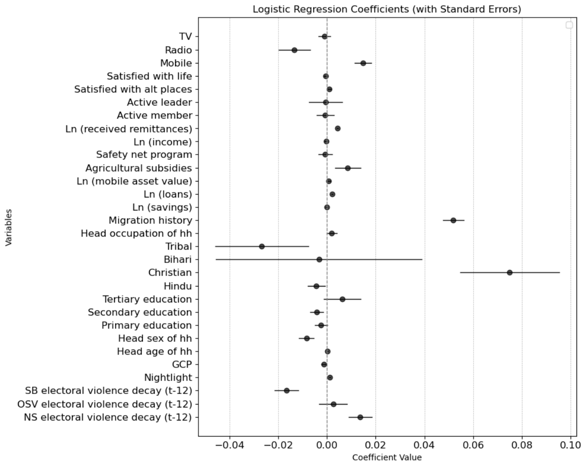

(d)

Figure S14: Logistic regression coefficients for *Violence* model including a) GED count of violent events, b) GED decay of violent events, c) count of electoral violence events and d) decay of electoral violence events.

and direction of the estimates for mobile access, received remittances, and migration history. The comparison also highlights additional advantages of relying on machine learning models by outlining the existence of non-linear relationships in the data visualised by the different colour patterns in Figure S16, and either fully hidden in the regression output (e.g. remittances) or resulting in non-significant effects (e.g. income).

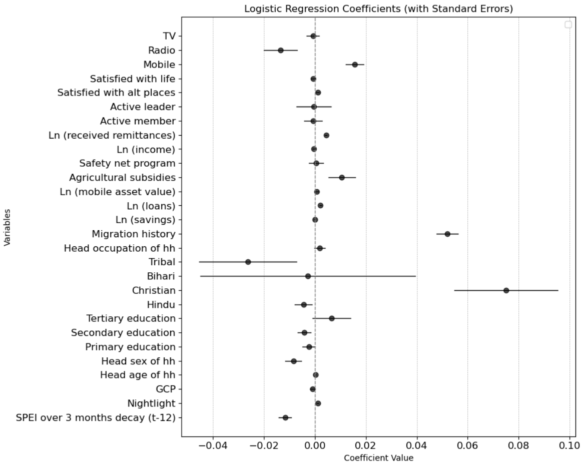

(a)

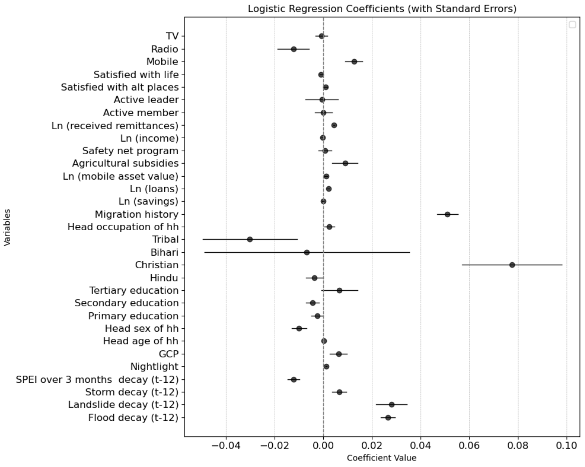

(b)

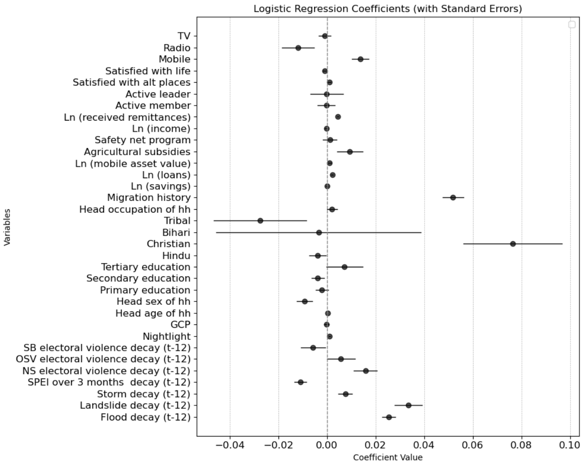

(c)

Figure S15: Logistic regression coefficients for *Disasters/Hazards* model including a) SPEI over 3 months (decay) and b) GDIS natural-hazard related disaster events (decay). Figure c) shows the logistic regression coefficients for *Violence & Disasters Hazards* model including decay of electoral violence events and SPEI over 3 months (decay) and GDIS natural-hazard related disaster events (decay).

Examining the features related to natural hazard-related disasters, we can observe similarities in terms of their significance and the directionality of their effects. The findings are, however, less clear when considering violent events. Interestingly, we see for example in Figure S5 d)

that one-sided election-related violence is not significant even if it shows the highest predictive importance in the machine learning models. Likewise, the regression output suggests a negative effect of state-based election-related violence whereas our primary analysis reveals the opposite trend.

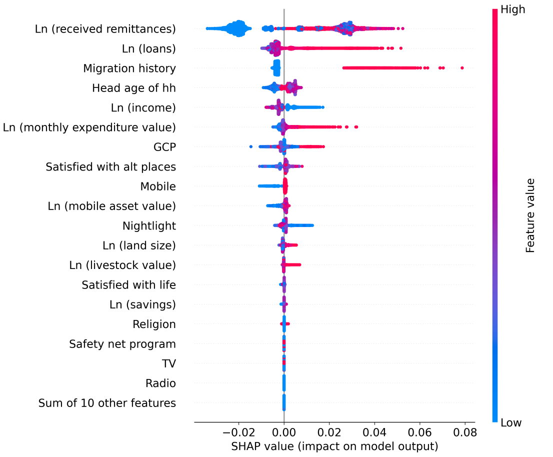

(a)

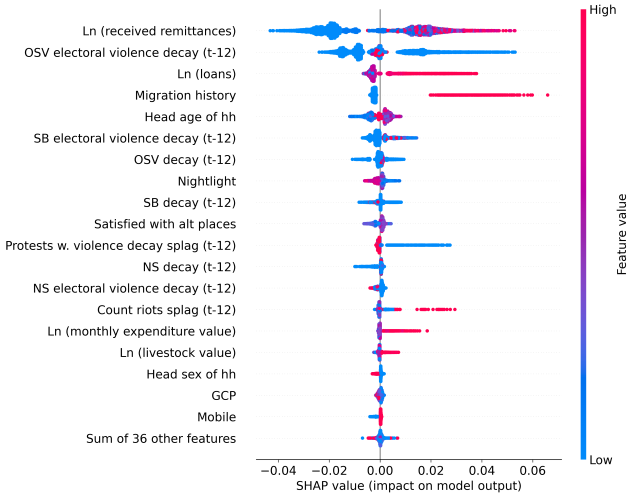

(b)

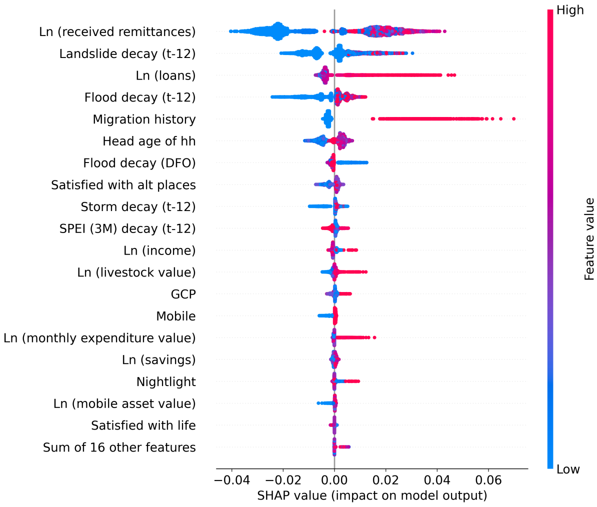

(c)

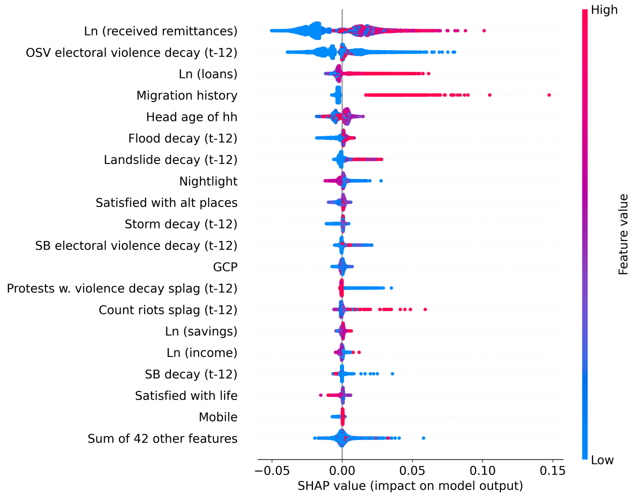

(d)

Figure S16: Summary plots of feature SHAP values, expressed in probabilities, for all models, including a) *Baseline*, b) *Violence*, c) *Disasters/Hazards* and d) *Full Distress* based on the best-performing out-of-sample performance. The x-axis shows for each feature the impact of each observation on the model output magnitude. The y-axis shows the feature names sorted by decreasing importance. The colour indicates the value of each observation, red indicating high values and blue low values.

Supplementary References

[1] Scott M. Lundberg, Gabriel Erion, Hugh Chen, Alex DeGrave, Jordan M. Prutkin, Bala Nair, Ronit Katz, Jonathan Himmelfarb, Nisha Bansal, and Su-In Lee. From local explanations to global understanding with explainable AI for trees. *Nature Machine Intelligence*, 2(1): 56–67, 2020.
